# Subcortical connections of area V4 in the macaque

**DOI:** 10.1002/cne.23513

**Published:** 2014-11-29

**Authors:** Ricardo Gattass, Thelma W Galkin, Robert Desimone, Leslie G Ungerleider

**Affiliations:** 1 Laboratory of Cognitive Physiology, Instituto de Biofísica Carlos Chagas Filho, UFRJ,Rio de Janeiro, RJ, 21941-900, Brazil; 2 Laboratory of Brain and Cognition, National Institute of Mental Health, National Institutes of Health,Bethesda, Maryland, 20892, USA; 3 Laboratory of Neuropsychology, National Institute of Mental Health, National Institutes of Health,Bethesda, Maryland, 20892, USA; 4 McGovern Institute, MIT,Cambridge, Massachusetts, 02139-4307, USA

**Keywords:** pulvinar, claustrum, caudate, putamen, superior colliculus, amygdala

## Abstract

Area V4 has numerous, topographically organized connections with multiple cortical areas, some of which are important for spatially organized visual processing, and others which seem important for spatial attention. Although the topographic organization of V4’s connections with other cortical areas has been established, the detailed topography of its connections with subcortical areas is unclear. We therefore injected retrograde and anterograde tracers in different topographical regions of V4 in nine macaques to determine the organization of its subcortical connections. The injection sites included representations ranging from the fovea to far peripheral eccentricities in both the upper and lower visual fields. The topographically organized connections of V4 included bidirectional connections with four subdivisions of the pulvinar, two subdivisions of the claustrum, and the interlaminar portions of the lateral geniculate nucleus, and efferent projections to the superficial and intermediate layers of the superior colliculus, the thalamic reticular nucleus, and the caudate nucleus. All of these structures have a possible role in spatial attention. The nontopographic, or converging, connections included bidirectional connections with the lateral nucleus of the amygdala, afferent inputs from the dorsal raphe, median raphe, locus coeruleus, ventral tegmentum and nucleus basalis of Meynert, and efferent projections to the putamen. Any role of these structures in attention may be less spatially specific. J. Comp. Neurol. 522:1941–1965, 2014.

Area V4 plays a central role in the relay of information from lower-order to higher-order visual cortical areas; in particular, V4 is a crucial link in the ventral processing stream for object recognition. In a previous study (Ungerleider et al., 2008), we reported that V4: 1) receives from and projects topographically back to early visual areas V2 and V3; 2) projects forward to superior temporal areas MT (see list for abbreviations) and FST, inferior temporal areas TEO, TEp and TEm, and parietal areas LIPv and LIPd; and 3) has intermediate-type connections with V3A, V4t, TEa, and FEF. In addition to these projections, V4 sites that represent eccentricities beyond 30° project to several additional visual areas in parietal cortex, namely, areas DP, 7a, PO, and VIP. The peripheral field representation of V4 also projects to area TF on the posterior parahippocampal gyrus. Overall, we found that central field representations of V4 have relatively stronger connections with ventral stream areas, whereas peripheral field representations of V4 have relatively stronger connections with dorsal stream areas. Some of these topographic connections likely mediate topographically organized sensory inputs to V4 (e.g., V1, V2), and others may mediate top-down inputs for spatial attention (e.g., FEF, LIP).

Although several reports have addressed the connectivity of the foveal and parafoveal representation of V4 with subcortical nuclei (Campos-Ortega and Hayhow, 1972; Benevento and Rezak, 1976; Benevento and Davis, 1977; Norden et al., 1978; Olson and Graybiel, 1980; Graham, 1982; Standage and Benevento, 1983; Rolls et al., 1983; Shipp and Zeki, 1985; Shipp, 2003), no study has yet addressed the full extent and topographic organization of the subcortical connections of V4. Thus, we decided to study the total extent of V4’s subcortical connections, as well as their topographic organization. Here we describe the subcortical connections of this area in nine macaque monkeys with combined tritiated amino acid (^3^H), wheat germ agglutinin conjugated to horseradish peroxidase (HRP), and retrograde fluorescent tracer injections placed under physiological control into 21 different retinotopic locations of V4. Because we were interested in delineating the complete set of connections of V4, our injections were large enough to include all eventual subregions within V4 at a given eccentricity.

## MATERIALS AND METHODS

All experimental procedures were approved by the NIMH Animal Care and Use Committee. The materials and methods are the same as those described previously (Ungerleider at al., 2008). ^3^H, HRP, and the fluorescent tracers fast blue (FB), diamidino yellow (DY), and bisbenzimide (Bis) were injected in 10 hemispheres of nine adult *Macaca mulatta*, weighing between 3.2 and 4.4 kg. In all animals, injections of tracers were placed into retinotopically specified sites (*n* = 21) in V4, which were determined by electrophysiological recordings (see Table1, Fig. 2). The injection sites, two or more in each animal, spanned eccentricities from central to peripheral vision in both the upper (*n* = 3) and lower (*n* = 18) visual fields (Gattass et al., 1988).

**Table 1:** Summary of Nuclei That Project to (P) and/or Receive From (R) V4

									Claustrum				Pulvinar														
Inj Site	Case	Tracer	Ecc	DR	MR	LC	VT	nbM	Ventral		Mid		P1		P2		P3		P4		Amyg (lb)		Cd	Put	SC	R	LGi
1	1c&p	^3^H	−2 to −16							t		–		t		t		t		t		–	t	t	t	t	–
4	8c	^3^H	−8							t		t		t		t		–		–		–	t	t	t	–	–
7	3c	^3^H	−3							t		t		t		t		t		t		–	t	t	t	t	t
8	9c	^3^H	−8							–		–		t		t		–		–		–	t	t	t	–	–
11	2p	^3^H	−16							t		–		t		t		t		t		–	t	t	t	–	–
12	4p	^3^H	−18							t		t		t		t		t		t		–	t	t	t	t	t
13	1p	^3^H	−22							t		–		t		t		t		t		–	t	t	t	t	t
17	7p	^3^H	−25							t		t		t		t		t		t		–	–	–	–	–	–
20	5p	^3^H	10							t		–		t		t		–		–		–	t	–	t	–	–
3	2c	HRP	−2	c	c	c	c	c	c	t	c	t	–	t	–	t	–	t	–	t	c	t	t	t	t	t	t
9	3p	HRP	−14	c	c	–	c	c	c	t	c	t	c	t	c	t	c	t	c	t	c	t	t	t	t	–	–
18	8p	HRP	−30	c	c	c	c	–	c	t	c	–	c	t	c	t	–	–	c	t	–	–	t	t	t	–	–
19	5c	HRP	4	c	c	c	c	–	c	t	c	t	c	t	c	t	–	t	c	t	–	–	t	t	t	–	–
21	6p	HRP	20	x	x	x	–	c	c	t	c	t	c	t	c	t	c	t	c	t	c	t	t	t	t	t	–
2	8c	FB	−1	–	–	–	–	–	c		c		c		c		–		–		–						
5	4c	FB	−2	–	–	–	–	–	c		c		c		c		c		–		–						
6	5c	FB	−2	–	–	–	–	–	c		c		c		c		c		–		–						
10	3p	Bis	−10	–	–	–	–	–	c		c		c		c		c		c		c						
14	7p	Bis	−25	–	–	–	–	c	c		c		c		c		c		c		c						
15	4p	DY	−18	–	–	–	–	–	c		c		c		c		c		c		–						
16	1p	Bis	−20	–	–	–	–	–	x		x		x		x		x		x		c						
c = cells																											
t = terminals																											

c, cells; t, terminals; gray areas, not applicable for the tracer; -, relevant sections with no label; x, relevant sections not analyzed; ECC, eccentricity; ^3^H, labeled amino acids.

### Receptive field recording

The experimental procedures for multi-unit recordings and cortical injections have been described in detail elsewhere (Desimone and Gross, 1979; Gattass and Gross, 1981; Gattass et al., 1987; Ungerleider et al., 2008). Briefly, prior to the first recording session, under ketamine and sodium pentobarbital anesthesia, the animal was implanted with a bolt for holding the head in the stereotaxic apparatus and a stainless steel recording chamber. In each recording session the animal was anesthetized with 2% halothane, followed by a 70:30% mixture of N_2_:O_2_. Muscular paralysis was induced by pancuronium bromide, and a respiratory pump connected to an endotracheal tube maintained artificial ventilation. The level of CO_2_, heart rate, and rectal temperature were continuously monitored and kept within the normal physiological range. The right eye was fitted with a contact lens, which focused the eye to the surface of a 57-cm radius translucent hemisphere placed in front of the animal. The locations of the fovea and the center of the optic disc were projected onto the hemisphere. The horizontal meridian was taken to be a line through both these points, and the vertical meridian an orthogonal line passing through the fovea.

Prior to the injections, we mapped the relevant portion of V4 with the aid of varnish-coated tungsten microelectrodes. The electrodes were assembled in a micromanipulator that could be used to record from small clusters of neurons or could hold a prealigned microsyringe to deliver the anatomical tracer. Visual receptive fields were plotted by moving white or colored bars onto the surface of the translucent hemisphere, under light-adapted conditions. Recordings continued until the desired visual field representation within V4 was located.

### Injections of V4

We injected anterograde and retrograde tracers under electrophysiological guidance into 21 sites in nine macaques. Pressure injections into the cortex were made using a 1-μl Hamilton syringe with a beveled 27G needle, which was guided into the appropriate site with the aid of an operating microscope. Sulcal and gyral landmarks were used to identify the location of area V4 (Zeki, 1978; Ungerleider and Desimone, 1986; Gattass et al., 1988). In six animals, injections were placed at physiologically determined sites on the prelunate gyrus under direct visualization of the cortex. In the remaining three animals, after the desired injection site was located electrophysiologically, a guide tube was advanced through the dura and placed about 300 μm above the intended injection site. The microelectrode was then advanced through the guide tube and the visuotopic location of the injection site was confirmed. The electrode was then withdrawn from the guide tube and replaced by a 1-μl Hamilton syringe. For the remainder of the article, we refer to each injection site as a case.

**Figure 1:**
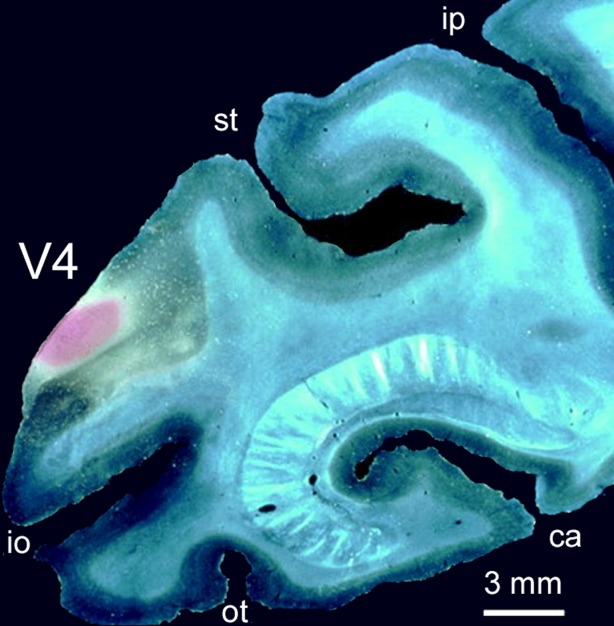
Photomicrograph of a coronal section of a representative case illustrating an injection into area V4, on the prelunate gyrus. See text for details.

In nine cases, we injected 0.15–0.3 μl of an equal-parts mixture of tritiated proline (New England Nuclear, Boston, MA; L-[2,3,4,5-3H], specific activity 100–140 Ci/mmol) and tritiated leucine (New England Nuclear L-[3,4,5-3H(N)], specific activity 100–140 Ci/mmol). The labeled amino acids, which had been evaporated and then reconstituted in 0.9% saline to give a final concentration of 50 μCi/μl, were injected at the rate of 0.02 μl/2 minutes. To minimize leakage of the tracer up the electrode track, the syringe was left in place for 30 minutes after the injection and then withdrawn into the guide tube, which was then removed from the brain. In seven cases, one to three injections (0.15–0.3 μl each at each site) of aqueous solutions of 2% FB, 4% DY, or 10% Bis were placed in a given area in the cortex. In five cases, two to four injections (0.2 μl each) of 5% wheat germ agglutinin conjugated to HRP were placed in V4. In the animals with injections involving both HRP and other tracers, the other tracer(s) were injected into given V4 sites in one procedure and, 4–6 days later, the HRP was injected into another site. A list of cases and tracers is shown in Table1 and an example of an injection is shown in Figure 1.

The amount, concentration, and liquid vehicle of the tracer injections as well as survival time were calculated to produce anterograde and retrograde labeling of equivalent size. However, the nature of tracers caused small differences in sensitivity. Among the tracers used, HRP was the most effective both as an anterograde and as a retrograde tracer. Among the fluorescent dyes, the most effective retrograde tracer was FB, which was closely followed by DY. Both were more effective than the other retrograde tracer (Bis).

For each case, a 2D “wire” map of the cortex was generated (Ungerleider and Desimone, 1986; Gattass et al., 1987). The 2D map was obtained by physically flattening the enlarged 3D wire models (10×) made with a thick wire through cortical layer 4 of the histological section and a thin wire connecting the sections at the borders and fundus of the sulci. The locations of the tracers, myeloarchitectonic borders, and recording sites were transferred onto the flattened maps.

**Figure 2:**
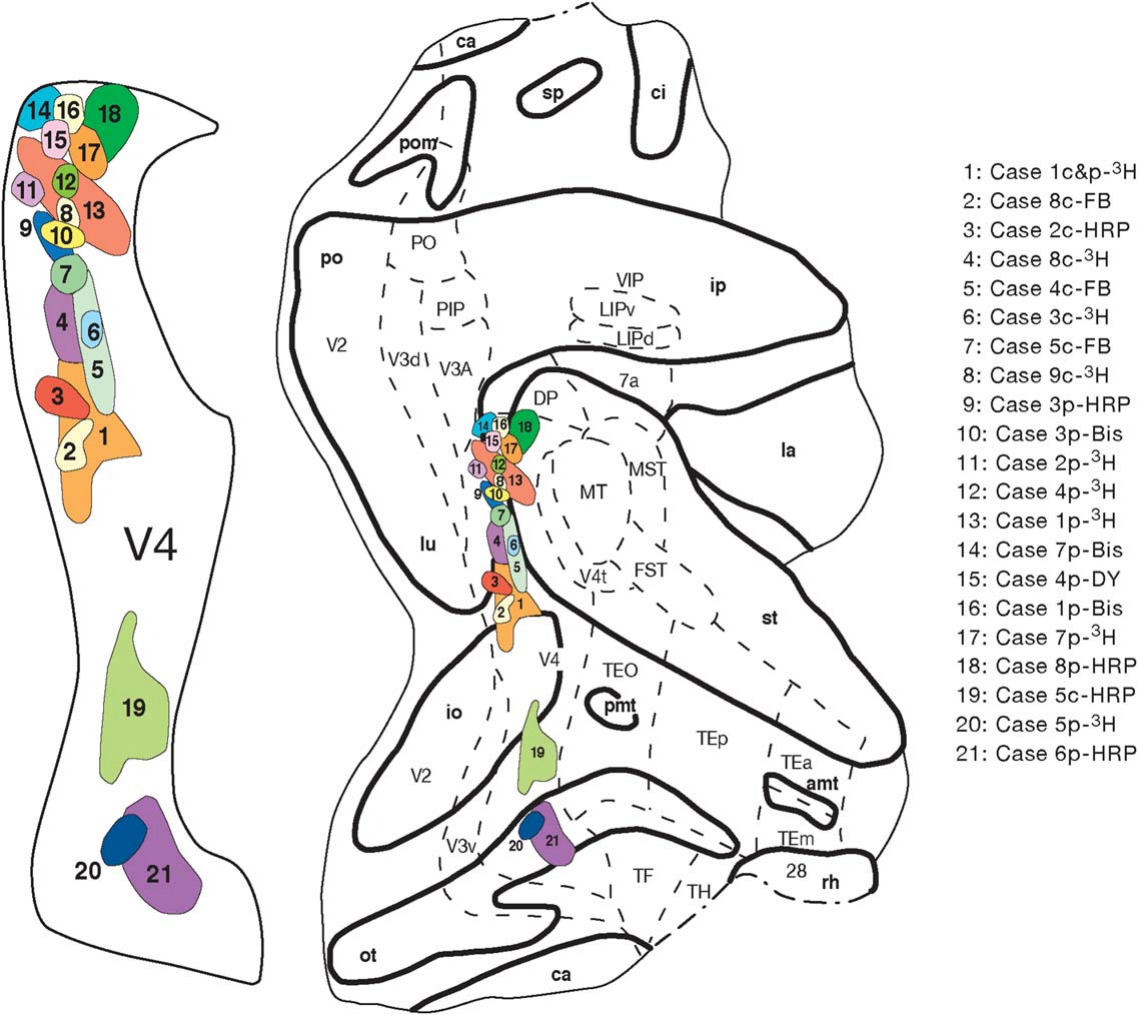
Injection sites in V4 shown in a flattened map of extrastriate cortex. Tracers were placed in 21 injections sites at central and peripheral locations in V4, in nine animals (cases) in 10 hemispheres. Each injection site is numbered and colored to match data from other figures. Myeloarchitectonic borders of visual areas are indicated with dashed lines. The injections from the individual cases were plotted on this map to best retain their locations relative to myeloarchitectonic borders and sulci. For names of areas and sulci, see abbreviations.

Figure 2 summarizes on a composed flattened map of extrastriate cortex the injection sites in area V4. The injections sites ranged from the fovea of V4 to eccentricities of 30° in the lower visual field and to eccentricities of 20° in the upper visual field. We only used injections that did not invade the white matter and showed consistent topographically organized connection with V2. In all cases, there were one or more labeled zones in V2 whose visuotopic locus was highly consistent with the visuotopic locus of the injection site in V4 (Gattass et al., 1988).

A comparison of the receptive fields recorded at the V4 injection sites with the estimated visual field representations of the locations of connections with V2 indicates a good agreement between the two loci (Gattass et al., 1981, 1988).

### Histological processing

After survival times of 2 days for HRP and 6–8 days for the other tracers, the animals received a lethal dose of sodium pentobarbital and were then perfused transcardially with 0.9% saline followed by 10% formaldehyde-saline. Their brains were blocked with the aid of a stereotaxic apparatus, removed from the skull, photographed, and stored in 30% sucrose in 10% formaldehyde-saline until they sank. Frozen sections, 33 μm in thickness, were cut in the frontal plane. One case (Case 6) was cut in the parasagittal plane. Every fifth section was mounted onto gelatinized slides, dehydrated, defatted, and processed for autoradiography according to the procedures of Cowan et al. (1972). These sections were dipped in Kodak NTB2 emulsion and exposed at 4°C for at least 12 weeks. Subsequently, the autoradiographs were developed in Kodak D19, fixed, and counterstained with thionin. Another series of sections 250 μm apart were processed for HRP histochemistry according to a modified tetramethylbenzidine protocol (Gibson et al., 1984). Of these sections, one in four (i.e., 1/mm) was counterstained with thionin, whereas the remainders were coverslipped unstained. Anterograde and retrograde labeling was plotted on enlarged photographs (10×) of the myelin-stained and/or the thionin-stained sections for subsequent analysis. The boundaries of the various thalamic nuclei were determined from the thionin-stained sections. The atlas of Olszewski (1952) was used as a reference for nomenclature and for delineating thalamic boundaries. The location of concentrations of silver grains, HRP-labeled cells and terminals, and fluorescent-labeled cells were assigned to specific subcortical structures in each animal and then combined to evaluate the topographical organization of each structure. Alternate sections were stained for myelin with the Gallyas’ (1979) method. The photomicrograph shown in Figure 1 was obtained with the aid of a Leitz Aristophot and a 5×7 color scanner. Contrast balance and the elimination of small scratch artifacts were done at the Photo and Arts Department of NIH, using Adobe Photoshop 7 (San Jose, CA).

## RESULTS

**Figure 3:**
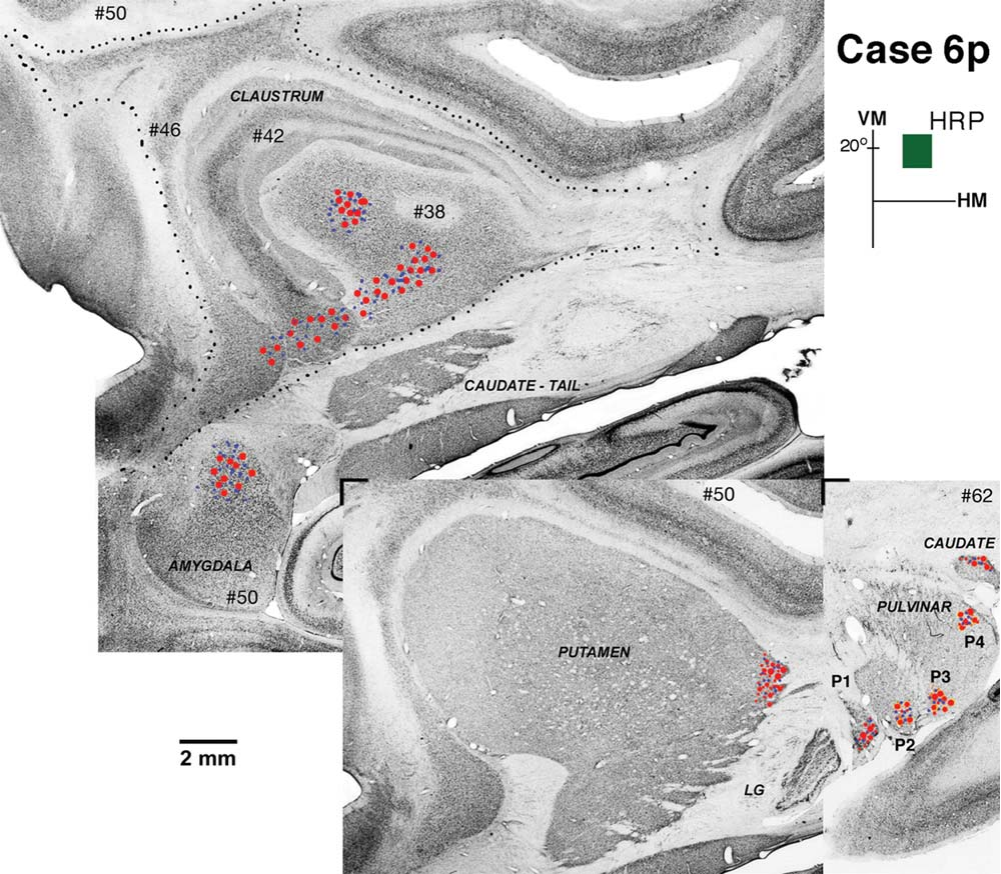
Connections of V4 with claustrum, amygdala, putamen, caudate, and pulvinar in a photomontage of parasagittal sections of Case 6p. Retrograde labeled cells (red-orange concentric icons) and/or terminals (blue dots) were found in each subcortical structure after injection of HRP in V4. Five parasagittal sections were cut, aligned, and staked to reconstruct most of the extent of the claustrum. An inset of section #50 shows the projection in the putamen (lower left), while another inset of section #62 shows the projections in caudate and in the different projection zones of the pulvinar (lower right). Two patches of labeled cells were found in in ventral (VC) and mid (MC) claustrum; three patches were found at corresponding topographical locations in P1, P2, P3, and P4 in the pulvinar; one patch in the putamen; one patch in the lateral basal (lb) nucleus of the amygdala and another in the caudate nucleus. For names of nuclei, see abbreviations.

The results are based on data from 21 injections of anterograde and retrograde tracers in V4 (Fig. 2). In the following sections we describe the topographic and nontopographic afferent, efferent, and reciprocal connections of V4. As described in detail below, V4 receives nontopographic projections from the dorsal and median raphe, locus coeruleus, ventral tegmentum, and the nucleus basalis of Meynert. V4 sends to and receives topographically organized projections from the pulvinar, claustrum, and interlaminar layers of the lateral geniculate nucleus, and nontopographic ones to and from the lateral basal nucleus of the amygdala. V4 also projects topographically to the superior colliculus and to the caudate, and nontopographically to the putamen and the thalamic reticular nucleus. Figure 3 illustrates in a single photomicrograph montage several subcortical sites connected with V4.

### Afferent connections of V4

#### Brainstem, midbrain, and forebrain structures: nontopographic inputs to V4

Injections of HRP in V4 revealed nontopographic inputs from the brain stem and midbrain structures. Cells were found in the nuclei of the monoamine ascending pathways, including a large number in the dorsal and median raphe, a smaller number in the locus coeruleus, and only a few in the ventral tegmentum. In the forebrain, two cases with injections of HRP in V4 and one with an injection of Bis also revealed labeled cells in the nucleus basalis of Meynert (see Table1).

### Reciprocal connections with V4

#### Pulvinar: topographic bidirectional connections

On the basis of electrophysiological recordings in the pulvinar, Bender (1981) described two separate fields, both of which are visuotopically organized. The first was termed the “PI” map, which is found mainly in rostrolateral PI, and extends into medial portions of adjacent PL. The second was termed the “PL” map, which partially surrounds the PI map and is located entirely in ventrolateral PL. Subsequently, Ungerleider et al. (1983, 1984) termed the PI and PL maps, respectively, the “P1” and “P2” fields based on connections of the pulvinar with V1 and MT. A third field, “P3,” was described by Ungerleider et al. (1984) based on its preferential connections with MT. It is located posteromedially in PI, but also includes small adjacent portions of PL and PM that lie dorsal to the brachium of the superior colliculus (see also Standage and Benevento, 1983). P3 does not seem to have a well-defined retinotopic map like its neighbor P1, although it has yet to be mapped electrophysiologically. Attempts to map P3 in *Cebus* anesthetized and paralyzed preparations were unsuccessful, inasmuch as most isolated units were unresponsive to visual stimulation (R. Gattass, unpubl. data). Dorsal to the P1–P3 fields, near the boundary between dorsal PL and PM, lies a region termed “Pdm” (Petersen et al., 1985; Robinson et al., 1986). Like P3, Pdm has little, if any, visuotopic organization. In the *Cebus*, there is a second visuotopic organization dorsal to P1, named Pμ by Gattass et al. (1978). In this study we use the term “P4” to describe the projection field of area V4 that is located dorsally to P1 and P2. P4 may be at least in part coextensive with Pdm (Petersen et al., 1985) and with Pμ (Gattass et al., 1978).

**Figure 4:**
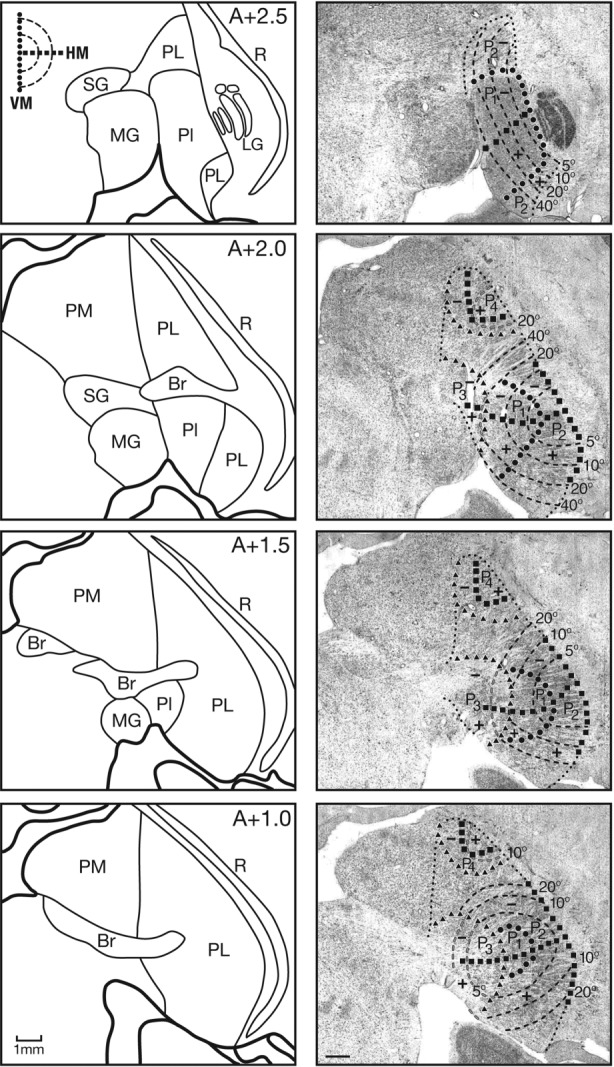
Representative coronal sections stained for Nissl through the rostral-to-caudal (top-to-bottom) extent of the pulvinar. Left: cytoarchitectonic subdivisions, according to Olszewski (1952). Right: the pulvinar fields, P1, P2, P3, and P4, are shown superimposed on each section. Solid circles indicate the representation of the vertical meridian, solid squares indicate the representation of the horizontal meridian, heavy dashes indicate isoeccentricity lines, gray colored dashes indicate isoeccentricity lines in areas of coarse topography, small solid triangles indicate the borders of P3 and P4, and small dotted lines indicate the borders of the pulvinar fields. The plus sign indicates the upper visual field representation and the minus sign indicates the lower visual field representation. The sections are spaced 0.5 mm apart, and they do not reach the caudal extent of the pulvinar. For names of nuclei, see abbreviations. Scale bars = 1 mm.

The relationship between P1–P4 and the architectonic subdivisions of the pulvinar are shown in Figure 4. The P1 and P2 visuotopic maps in the pulvinar were charted onto Nissl-stained sections based on previously published work by Bender (1981) and Ungerleider et al. (1983, 1984). The P3 map was similarly charted. The first estimate of P3’s borders was guided by Ungerleider et al. (1984) and then by the distribution of label in the current V4 cases. The dorsal border of P3 (i.e., the portion above the brachium of the superior colliculus) was adjusted to be compatible with the distribution of calbindin immunoreactivity presented in previous work (Adams et al., 2000) and with the distribution of cells and terminals in the current V4 cases. The estimate of P4’s border was guided by Adams et al. (2000) and then by the distribution of label in the current V4 cases. Thus, our assignment of cells and terminals to P1–P4 is based on estimated borders of these regions.

**Figure 5:**
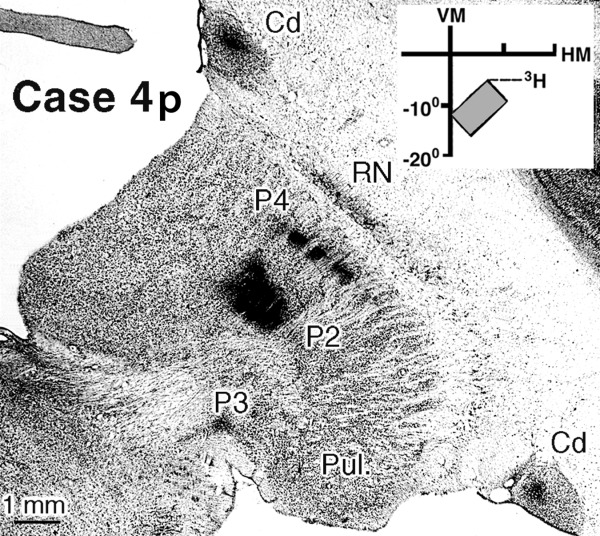
Photomicrograph of the pulvinar and surrounding areas showing the projections from V4 in Case 4p. Three patches of silver grains were found at corresponding topographic locations in P2, P3, and P4 of the pulvinar; one patch was found in the thalamic reticular nucleus (RN) and two additional patches were found in the caudate nucleus (Cd).

**Figure 6:**
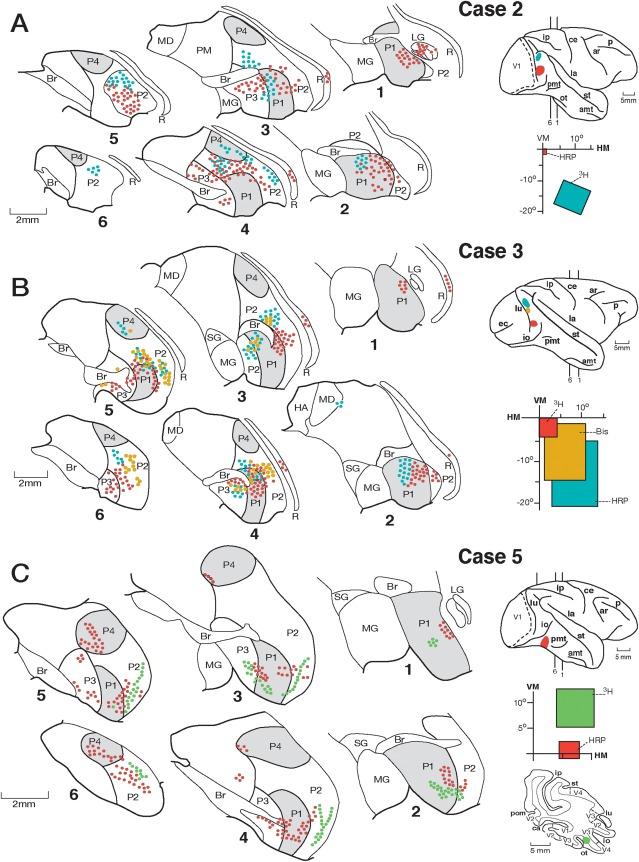
Connections of pulvinar with V4 in Cases 2, 3, and 5. Two or three anterograde and retrograde tracers were injected at topographical locations (right) in V4 as illustrated in the lateral view of the hemisphere. Labeled cells and terminals are shown in coronal sections through the pulvinar and surrounding areas (left). For details see text.

**Figure 7:**
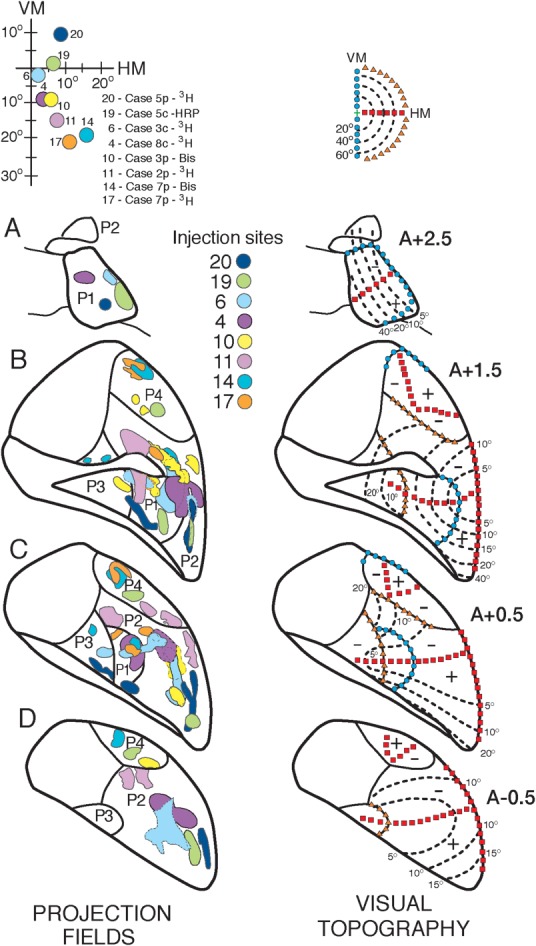
Four topographically organized projection fields (P1–P4) of the pulvinar revealed after injections of tracers in V4 at the eccentricities shown in eight selected cases (left). A–D: Reconstructions of coronal sections of the pulvinar from anterior (A) to posterior (D) regions. Representations of the topographic maps in the four projection zones of the pulvinar are drawn at four coronal sections through the pulvinar (right).

Several clusters of labeled cells and terminals were found in the pulvinar after injections of retrograde and anterograde tracers in V4 at different topographic locations. Examples of such data are illustrated in Figures 7, where labeled cells and terminals were found in the projection zones P1, P2, and P3, as defined previously (Ungerleider et al., 1983, 1984). These labeled cells and terminals were not restricted to these projections fields, however, but extended dorsal to the field that we term P4 (Figs. 5–7). As shown in Figure 5, the distribution of labeled cells appeared in small clusters in PI, part of PL, and to a lesser extent in PM. The borders of these small clusters appeared to coincide with the limits of the chemoarchitectonic subdivisions of the pulvinar (see Adams et al., 2000); however, because the tissue from our brains was processed many years ago, no direct comparison between chemoarchitecture and projections was possible in our study.

The projections to the pulvinar are illustrated in parasagittal (Fig. 3) and coronal (Fig. 5) sections. Figure 3 shows projecting cells and terminals in a montage of parasagittal sections after an injection in the upper visual field of V4. Four patches of cells and terminals, corresponding to the P1, P2, P3, and P4 fields, are shown in section 62. Note that the patches corresponding to an injection in the upper field are located ventrally in fields P1–P3 and dorsally in P4. Figure 4 shows a photomicrograph of a coronal section of the pulvinar and surrounding areas illustrating the projections from the lower visual field of V4 (Case 4p). Three patches of silver grains were found at corresponding topographic locations in P2, P3, and P4; note that the patches are located dorsally in P2 and P3 and ventrally (i.e., away from its dorsal border) in P4. Additional patches were found in the thalamic reticular nucleus and both dorsally and ventrally in the caudate nucleus.

Figure 5 shows the distribution of labeled cells and terminals in the pulvinar and surrounding areas after injections of anterograde and retrograde tracers (HRP, Bis, and ^3^H) in V4 in Cases 2, 3, and 5. The injections in Case 2 (Fig. 6A) were placed on the lateral convexity of the prelunate gyrus in V4’s lower visual field representation of the fovea (HRP) and at about 15° eccentricity (^3^H). The anterograde projections from V4 extended from the anterior portion of PI (section 1) to the posterior portion of the PL (section 6). These projections encompass the P1 and P2 projection fields (sections 1–6), P3 (sections 3 and 4), and P4 (section 4). Projections were also seen in the interlaminar zones of the lateral geniculate nucleus and in the thalamic reticular nucleus (see also Fig. 5). Figure 6B shows the distribution of labeled cells and terminals in the pulvinar and surrounding areas after injections of an anterograde tracer (^3^H), a retrograde tracer (Bis) and a bidirectional tracer (HRP) in central (2° eccentricity), intermediate (8° eccentricity), and peripheral (14° eccentricity) representations of V4’s lower visual field in Case 3. Several clusters of labeled cells and terminals were found in P1 (sections 1–4), P2 (sections 2–6), and P3 (sections 3–6). Labeling was also found in P4, in the dorsal portion of PL (section 5). Figure 6C shows the distribution of labeled cells and terminals in the pulvinar and surrounding areas after injections of an anterograde (^3^H) and a bidirectional (HRP) tracer in central (4° eccentricity) and intermediate (10° eccentricity) representations of V4’s upper visual field in Case 5. Several clusters of labeled cells and terminals were found in P1 (sections 1–5), P2 (sections 2–6), P3 (sections 3–5) and P4 (sections 3–6). Unlike the previews cases, the projections in Case 5 were found in more ventral portions of P1, P2, and P3.

Figure 7 shows a summary of the regions containing labeled cells and terminals after injections in V4 in eight selected cases, and the inferred visuotopic organization of the pulvinar with well-defined topographic maps in P1 and P2, and cruder maps in P3 and P4, which have some separation of the upper and lower field projection sites. The projections to and from V4 in these eight cases encompass almost the entire extent of the P1–P4 fields of the pulvinar. The injection sites 20 and 19, located in the upper field representation of V4, led to ventral patches in P1 (Fig. 6A–C), P2 (Fig. 6B–D), and P3 (Fig. 6C), and to a central patch in P4 (Fig. 6B–D). The injections in the lower field representation of V4 led to dorsal patches in P1 (Fig. 6B,C), P2 (Fig. 6B–D), and P3 (Fig. 6B,C), and both dorsal and ventral patches in P4 (Fig. 6B,C). The patches revealed by the 20 injections in V4 show a considerable overlap in all pulvinar fields, suggesting coarser topographic organizations in these fields and/or larger receptive fields in the pulvinar when compared with those in V4.

#### Claustrum: coarse topographic bidirectional connections

**Figure 8:**
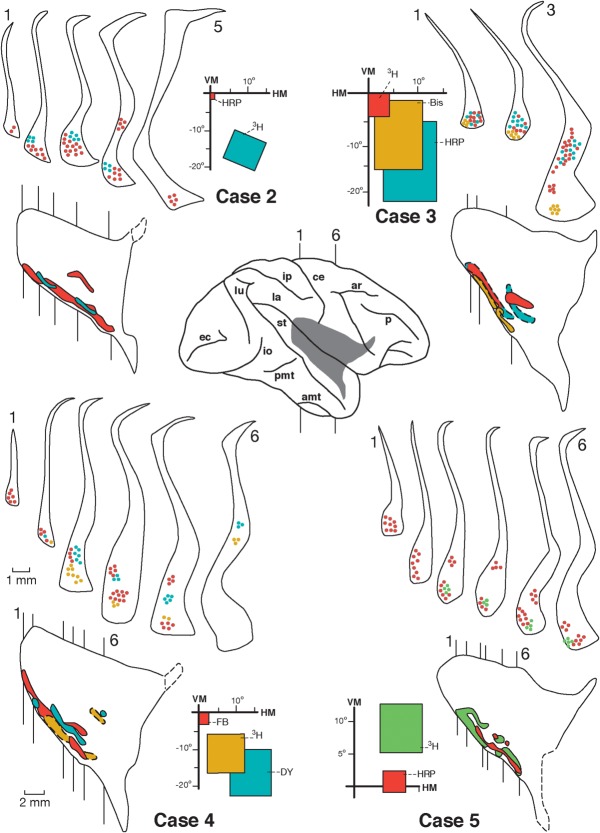
Connections of the claustrum to and from area V4. Afferent and efferent connections of V4 to the claustrum are shown in coronal sections at the level indicated in the lateral reconstruction of the claustrum in four selected cases. The projections of one lateral reconstruction of the claustrum onto the lateral reconstruction of the hemisphere are shown in gray (center).

**Figure 9:**
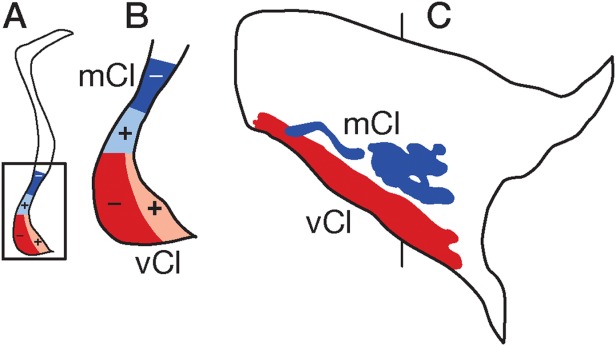
Two topographically organized areas in the claustrum: vCL (blue) and mCL (red). (+), representation of the upper visual field; (–), representation of the lower visual field in the areas of the claustrum.

The claustrum is a very narrow nucleus that wraps around the thalamus laterally. It resembles a leaf with two segments, one extending anteriorly, into the frontal lobe and the other extending anteriorly into the temporal lobe. A lateral reconstruction of the claustrum reveals that this nucleus is surprisingly large in its anterior-to-posterior extent. Reciprocal connections with the claustrum in four cases with V4 injections are shown in Figure 8. In the center of the figure, the lateral reconstruction of the claustrum is shown on a lateral view of the right hemisphere. In the four corner panels, coronal sections through the claustrum and their lateral reconstructions show the location of labeled cells and terminals in two small regions: a larger one located in the ventral portion of the claustrum, and a smaller one located more dorsally in its mid portion. The case illustrated in the lower right corner (Case 5) received injections in the representation of V4’s upper visual field, whereas the other cases illustrated received injections in V4’s central representation and lower visual field. The connections found in the two regions of the claustrum were very reliable in all animals studied. We named these regions the ventral claustrum (vCl) and the mid claustrum (mCl), respectively. After injections in V4, both of the regions labeled in the claustrum (containing both cells and terminals) were elongated in the anterior-to-posterior dimension. Both appeared to have a coarse topographic organization. In the more dorsal region, labeled mCl, the connections with V4’s lower visual field were dorsal to the connections with V4’s upper visual field (Fig. 9). In the ventral region, labeled vCl, the visuotopic organization was less clear but there was a tendency for the connections with V4’s upper visual field to be located medial to the connections with V4’s lower visual field.

#### Interlaminar LGN: “topographic bidirectional” connections

Injections in V4 with anterograde tracers resulted in labeled terminals in the interlaminar layers of the lateral geniculate nucleus (LGNi) in four of nine cases (see Case 2, Fig. 4), but injections with retrograde cases did not result in labeled cells in any case. No labeled cells were found in the S layer, as previously described by one of the authors (Soares et al., 2001a). We considered the LGNi as having bidirectional connections with V4 because direct projections from the koniocellular layers of the LGN to area V4 have been reported previously (Wong-Riley, 1976; Benevento and Yoshida, 1981; Fires, 1981; Yoshida and Benevento, 1981; Yukie and Iwai, 1981; Bullier and Kennedy, 1983; Soares et al., 2001a). One possible explanation is for why we were unable to see those labeled cells is that the fluorescent plotting of subcortical structures was done after the plotting of the cortical-cortical projections. The LGNi cells are very small, so by the time we plotted the subcortical projections the fluorescence of these cells may have faded, preventing their being seen.

#### Amygdala: nontopographic bidirectional connections

**Figure 10:**
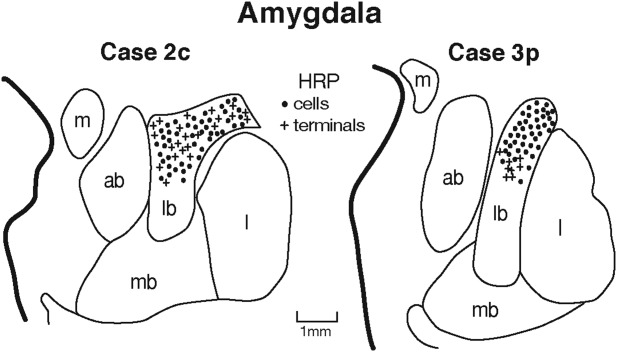
Nontopographic connections to the lateral basal nucleus of the amygdale (lb). Cells (circle) and terminals (+) in the dorsal part of the amygdala after HRP injections in V4 are shown in one coronal section in two representative colors.

In three of the five cases with injections of HRP in V4, a large number of labeled cells, and a smaller number of terminals, were observed in the dorsal portion of the lateral basal nucleus (lb) of the amygdala. Figure 10 shows the location of these labeled cells and terminals in coronal section in Cases 2 and 3. Figure 3 also shows intermingled inputs to and outputs from V4 in Case 6. Amygdala connections did not show any topographic organization, but our data for this subcortical structure are limited. None of the other cases showed any labeling in the amygdala.

### Efferent connections of V4

#### Putamen: nontopographic V4 projections

Projections from V4 to the posterior portion of the putamen (Fig. 2), which were found in 12 of 14 cases (Table1), did not follow any topographic pattern. The projection zone was in the most caudal part of the putamen, where this nucleus appears as segmented islands in coronal section. In all cases, the projections extended from the dorsal to the ventral portion at this most caudal level of the nucleus. Figure 10 illustrates, in a lateral reconstruction of the putamen, the entire efferent projection zone from V4, as well as the entire projection zone from V4’s central visual field injections and that from V4’s peripheral visual field injections (Fig. 1A,B). This figure also shows projections in selected coronal sections from three representative cases (Fig. 1C) as well as the projections in lateral reconstructions of the putamen (Fig. 1D,E).

**Figure 11:**
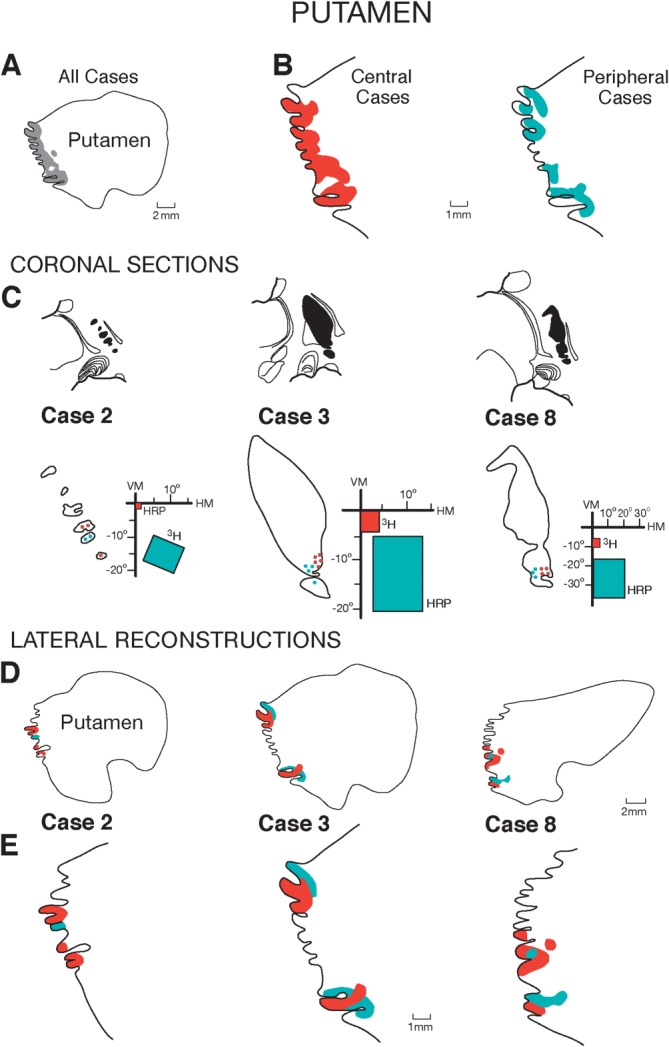
Projections to the putamen. Labeled terminals were found in different topographical locations in the putamen. A: superimposition of projection zones all animals (left) segregated in upper and lower field projections (right) B: coronal sections through the putamen in three animals. The locations of these cells are shown in the lateral reconstructions (C). The labeled cells are located in the posterior portion of the putamen in all animals as illustrated in the enlarged view (D) of the posterior portion of the putamen.

#### Caudate nucleus: V4 projections

The caudate nucleus is a long horseshoe-shaped structure, which has been subdivided anatomically into the head, body, genu, and tail. Of the 14 cases with anterograde tracers, 13 showed label in the caudate (Table1). Figure 2 shows the projections from V4 to the caudate nucleus across all these cases, superimposed on lateral and dorsal reconstructions of the nucleus (Fig. 2A,B) and on selected coronal sections (Fig. 2C). Projection from V4 to the caudate included mainly the body, extending posteriorly into the genu and the posterior two-thirds of the tail, as well as anteriorly into the dorsolateral portion of the head. Ventromedial portions of the head and the most anterior portion of the tail were free of label.

**Figure 12:**
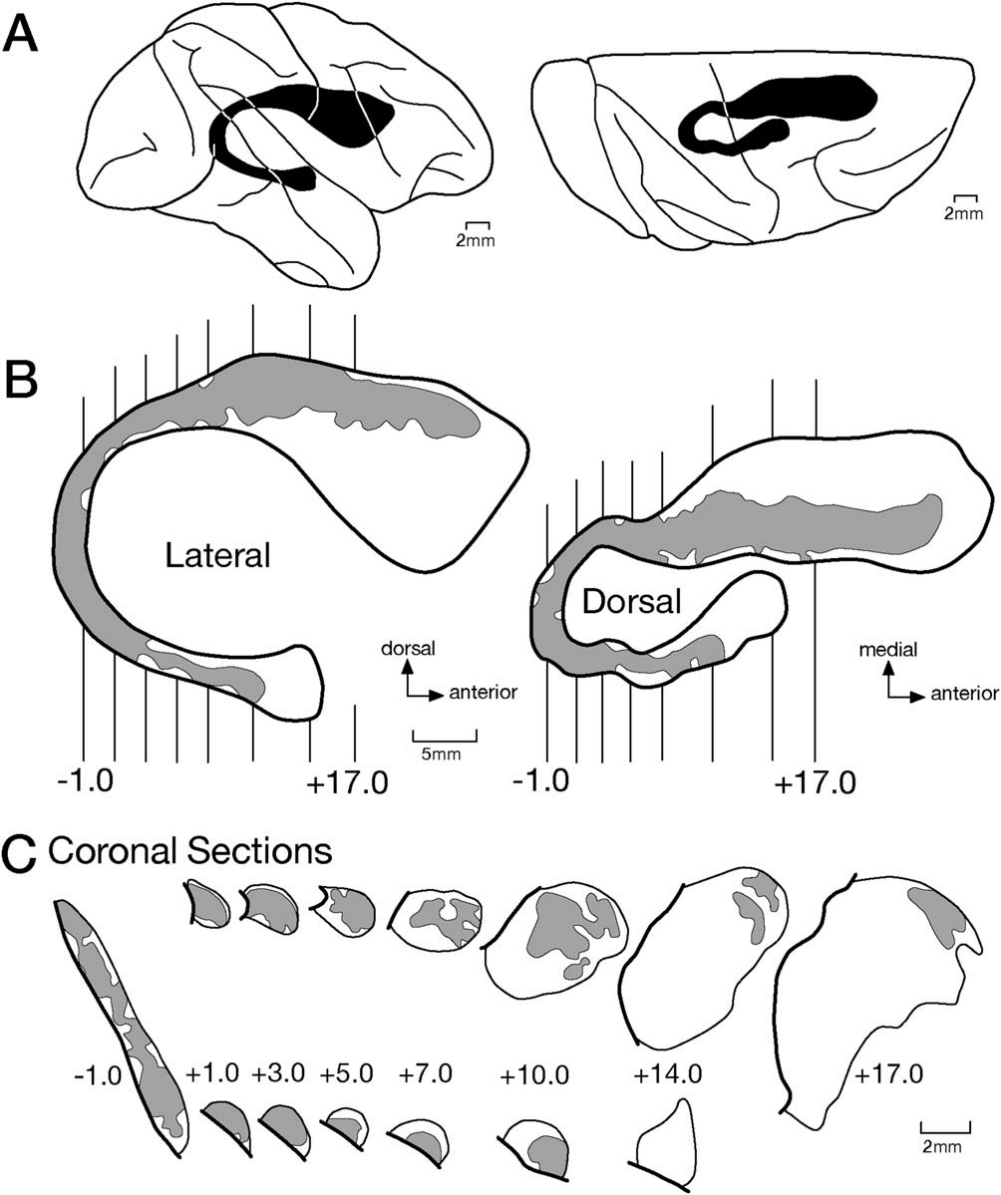
Projections to the caudate nucleus. The entire projection of V4 to the caudate nucleus (gray areas) in all animals encompasses most of the caudate, except for the most anterior portions of the head and the tail of the nucleus. The reconstructions of the projections in the caudate are shown on the representation of the lateral and dorsal hemispheres (top). The projection zones were superimposed onto the lateral and dorsal reconstructions of the caudate (middle) and onto coronal section through the caudate nuclei (bottom).

Figure 3 illustrates projections to the caudate from V4’s foveal representation and from both lower and upper visual field representations. In Case 2, injections of anterograde tracers were placed in the foveal and peripheral representations of V4’s lower visual field. Despite the fact that the injections were located in different representations of the visual field and occupied very separated areas on the prelunate gyrus, the resultant labeling was intermingled (see coronal sections). However, in most instances the projections from two nonoverlapping injections formed alternating or interleaved bands across anterior-to-posterior levels; Cases 8 and 5 (Fig. 2B,C) show this interleaved pattern. Compared to injections in the lower visual field representation of V4 (see Cases 2 and 8), injections placed in V4’s upper visual field (see Case 5) revealed projections located somewhat more ventrally in the body of the caudate and anteriorly in the tail (Fig. 1C), suggesting a possible crude topography based on proximity, with ventral V4 projecting more ventrally in the body and anteriorly in the tail compared to dorsal V4. Thus, the caudate nucleus appears to have, at a local scale, a topographically intermingled mosaic organization but at a coarser scale, a crude visual topography.

**Figure 13:**
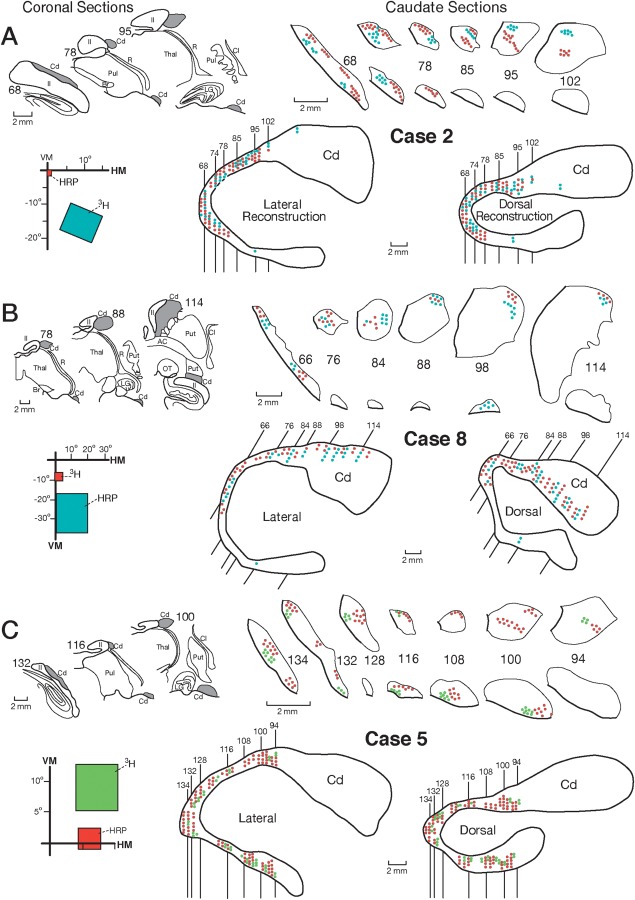
Projections to the caudate nucleus in Cases 2, 8, and 5. Terminals were found in the caudate nuclei after injections of two anterograde tracers in V4. The levels of the coronal sections (upper left) are indicated by vertical lines in the reconstructions of the caudate. In the enlarged sections (upper right) of the caudate nuclei, the location of terminals are shown after injections of ^3^H-amino acids and HRP at different topographical locations. The labels are also shown in the lateral (left) and dorsal (right) reconstructions of the caudate nucleus.

#### Superior colliculus: topographic V4 projections

In 13 of the 14 cases with anterograde tracers injected in V4, projections were found in the upper layers of the superior colliculus (SC), which followed a topographic pattern compatible with the known visuotopic map of the macaque colliculus (Cynader and Berman, 1972; Tabareau et al., 2007). According to Cynader and Berman (1972), the fovea is represented anteriorly, the peripheral visual field posteriorly, the lower visual field laterally, and the upper visual field medially. As illustrated in Figure 4 (see both coronal sections and dorsal view of the colliculus), the location of the labeling in the colliculus after V4 injections was consistent with this visuotopic organization. Central field injections showed projections in the anterior portion of the colliculus (Cases 3 and 6), whereas peripheral field injections showed projections in more posterior locations (Cases 11, 13, and 18). Lower field injections showed projections in the lateral portion of the colliculus (Cases 1, 4, and 9), whereas upper field injections showed projections in more medial locations (Cases 19 and 20). Thus, the projections from V4 are in topographic register with the visuotopic organization of the superior colliculus. We did observe, however, that in some cases the projection zone appeared to be elongated in the anterior-to-posterior plane compared to what one would expect from the receptive field recordings (e.g., see Cases 3, 12, and 18; Fig. 3); the reason is currently unclear. In all cases, projections to the colliculus from V4 extended ventrally from the stratus griseum superficiale to include, although less densely, the stratus opticum.

**Figure 14:**
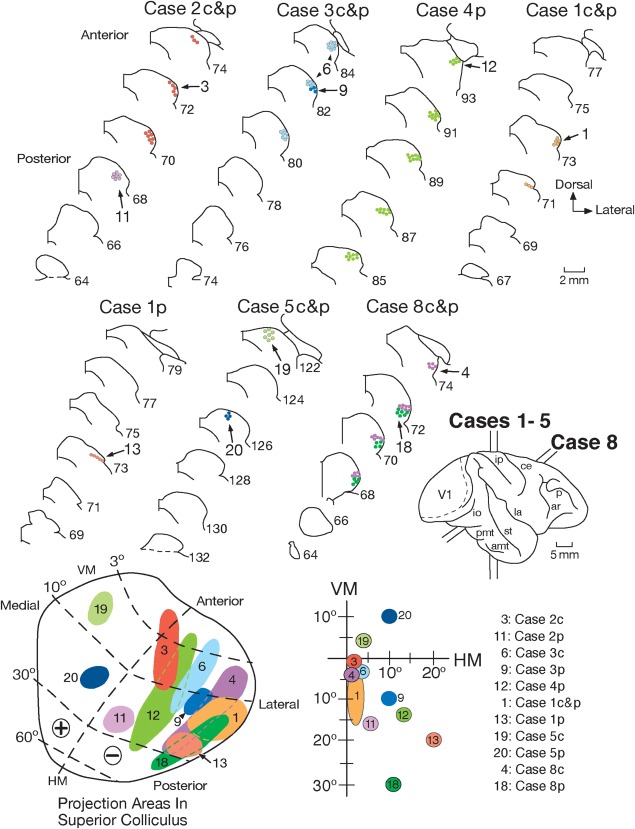
Topographically organized projections of the V4 to the superficial layers of the superior colliculus in six cases. Coronal (Cases 1–5) and oblique (Case 8) sections of the superior colliculus show topographically organized projections, after injection of one or two anterograde tracers in V4. The visuotopic locations of the injections sites are shown in the representation of the contralateral visual hemifield (lower right) and the reconstruction of the projection zones are shown on the superior colliculus surface (lower left).

**Figure 15:**
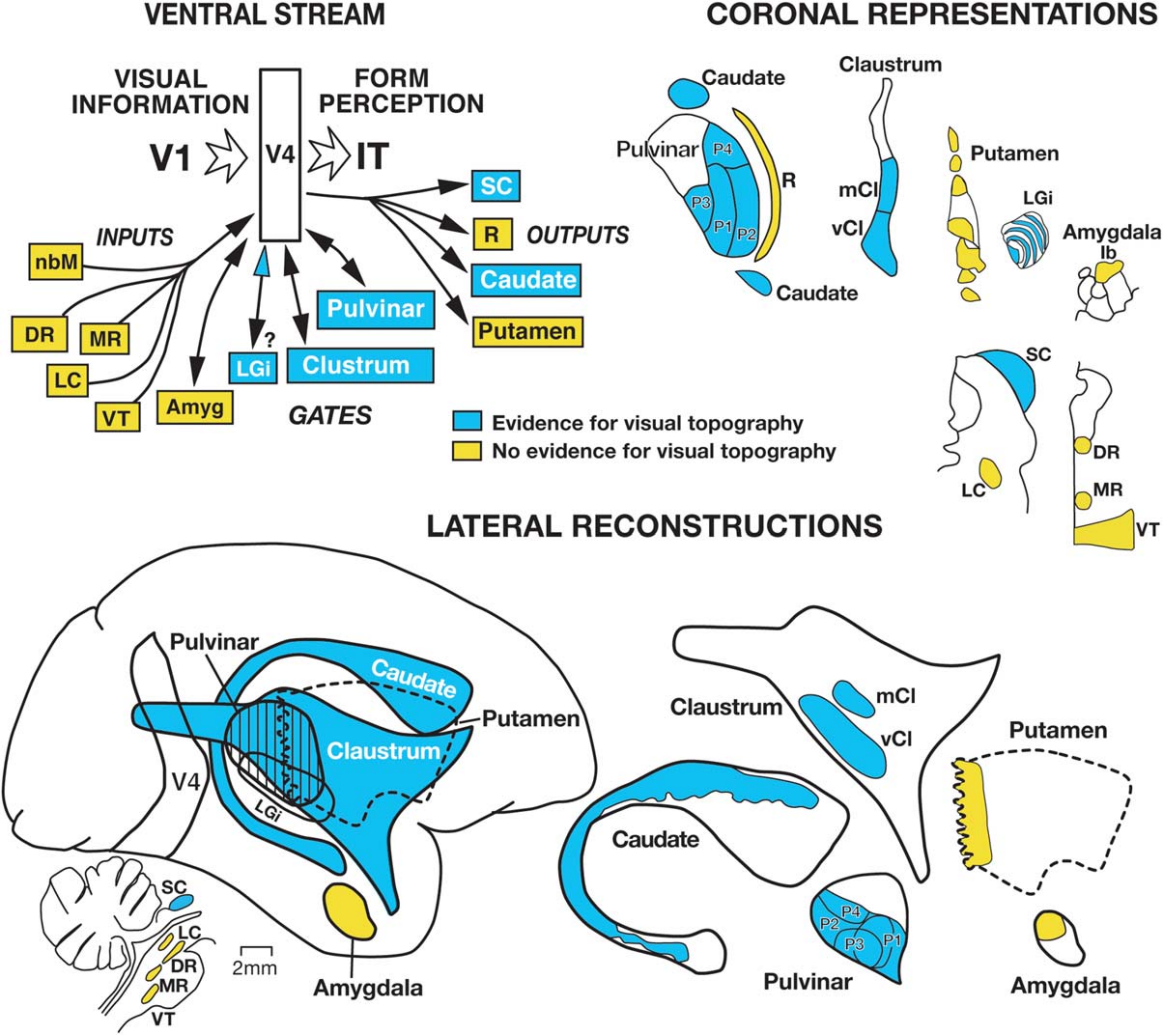
V4’s subcortical connections can be segregated into visutopically organized (blue) and non-visuotopically organized ones (yellow). Topographical gates (and efferents) allow spatial attention filtering of the information leading to the timporal lobe. Together these structures could act as topographically distributed networks, enhancing or facilitating visual processing at specific loci in V4.

#### Thalamic reticular nucleus: V4 projections

Injections in V4 resulted in terminals in the thalamic reticular nucleus in 6 of the 14 anterograde cases (see Cases 2 and 3 in Fig. 4; see also Table1). In most instances, the location of the label in the reticular nucleus was adjacent to the labeled region in the pulvinar of that case (see Fig. 5).

## DISCUSSION

Extrastriate area V4 plays a key role in relaying information from V2 to higher-order areas in the inferior temporal cortex (areas TEO and TE) that are critical for object recognition (Ungerleider at al., 2008). Topographically organized, sensory inputs to V4 are modulated by mechanisms for spatial attention (see Baluch and Itti, 2011, for a recent review), which likely involve top-down feedback from parietal and prefrontal areas (Posner, 1980; Rafal and Posner, 1987; Posner and Petersen, 1990; Kastner et al., 1998; Kastner and Ungerleider, 2000; Schafer and Moore, 2011). Subcortical contributions to mechanisms for spatial attention in V4 are not well understood, but may involve both the superior colliculus and the pulvinar (Petersen et al., 1985; Desimone et al., 1990; Karnath et al., 2002; Carello and Krauzlis, 2004; Hafed and Krauzlis, 2008; Snow et al., 2009; Saalmann and Kastner, 2011), both of which have visuotopic organizations. In the present study, we therefore mapped the full extent and topographic organization of the subcortical connections of V4.

Our findings indicate that V4’s subcortical connections can be grouped into three categories. The first category comprises afferent structures that project to V4; these include nuclei within the midbrain and forebrain. The second category comprises structures that have topographic or nontopographic reciprocal (bidirectional) connections with V4; these include four subdivisions of the pulvinar, two portions of the claustrum, the LGNi, and a restricted region within the lateral basal nucleus of the amygdala. The third category comprises structures that receive topographic or nontopographic efferents from V4; these include the superior colliculus, the thalamic reticular nucleus, a large extent of the caudate nucleus, the most caudal portion of the putamen, and the intralaminar layers of the lateral geniculate nucleus. Together, these subcortical structures could act as topographically distributed networks, enhancing or facilitating visual processing at specific visual field loci within V4, or could allow for the establishment of visuomotor habits.

### Afferents to V4

#### Brainstem and midbrain

In almost all cases with HRP injections in V4, we observed retrogradely labeled cells in the dorsal and median raphe, locus coeruleus, and ventral tegmentum. Both the location and morphology of these cells correspond to the monoamine-containing neurons in the brainstem and midbrain (Felten and Sladek, 1983). Together, these structures are part of circuits that provide diffuse activation of the cerebral cortex (Wu et al., 2007; Jones, 2008). Presumably, the projections from the locus coeruleus to V4 provide noradrenergic input, while projections from the ventral tegmentum and from the dorsal and median raphe provide dopaminergic and serotonergic input, respectively (Gatter and Powell, 1977; Mason and Fibiger, 1979; Porrino and Goldman-Rakic, 1982; Tigges et al., 1982; Wilson and Molliver, 1991).

#### Basal nucleus of Meynert

The basal nucleus of Meynert (nbM) is a group of cells in the substantia innominata of the basal forebrain that has widespread projections to the cortex (Mesulam and Van Hoesen, 1976; Wenk et al., 1980; Mesulam et al., 1983) and is rich in acetylcholine and choline acetyltransferase. Several of our cases with HRP injections showed retrograde label in approximately the same region of the nbM, consistent with its widespread cortical projection field. The location of the label appeared to lie within the nbM’s intermediate subdivision, in a similar location to that observed after injections of retrograde tracers into macaque inferior temporal cortical areas TEO and TE (Webster et al., 1993).

### Bidirectional connections with V4

#### Pulvinar

Adams et al. (2000) showed that projections from the pulvinar to V1 and V2 in macaque are overlapping in two separate fields that are in register with the visual field maps of P1 and P2. In some cases, an additional projection was found from P3 to area V2. MT projecting cells were also found in P1 and P2, but were mainly concentrated in the most medial portion of P3. The projections of the pulvinar to V4 were found in the ventral portion of PL and, less intensely, in the caudal portion of PI (Baleydier and Morel, 1992). Adams et al. (2000) showed an extensive projection zone to V4 from the region named P2, with sparser projections from P1 and still sparser from P3. Our current results showed that V4 projecting neurons are located in the central portion of PL, similar to the projections to V2 described above, as well as in the dorsal portion of PL, named P4 here.

Immunohistochemical studies in macaque, capuchin, and squirrel monkeys have revealed five similar subdivisions of the pulvinar, which include all of the inferior pulvinar, but which also encompass parts of the lateral and medial pulvinar, named PIP, PIM, PIC, PIL, and PILS (Cusick et al., 1993; Gutierrez et al., 1995; Gray et al., 1999; Adams et al., 2000; Soares et al., 2001b). The similarities in the chemoarchitectonic subdivisions contrast with the distinct connectivities and the different visuotopic organizations found in the pulvinar. In *Cebus,* Soares et al. (2001b) were unable to clearly segregate P1 from P2 based on the connectivity with V1, V2, MT, and V4, in spite of great similarities of the chemoarchitecture in *Macaca* and *Cebus*. Areas V2 and V4 in *Cebus* have preferential connections with P1, which may correspond to the ventrolateral complex of the *Cebus* (Gattass et al., 1978), and would correspond to both P1 and P2 of *Macaca*. A similar segregation was described by Cusick et al. (1993) and Stepniewska and Kaas (1997), who also established that the subdivisions of PI that receive ascending connections from the superior colliculus are distinct from the portion of the nucleus that projects to area MT. Adams et al. (2000) showed that the connections of V4 and MT are segregated into different chemoarchitectonic divisions. They suggested that the thalamic integration of cortical afferents and efferents could take advantage of the lamellar organization of the chemoarchitectonic divisions, where superimposed concentric shells are aligned through its visuotopic organization. This “onion”-like structure would allow local topographic integration necessary for spatial visual enhancement or suppression of specific visual information. Inasmuch as the inferior pulvinar (P1, P2, and P3) is the only tecto-recipient region of the pulvinar (Partlow et al., 1977), the function of its connections with V4 is probably to modulate tectal input to V4.

Kaas and Lyon (2007) have further proposed that the pulvinar nuclei could be segregated into two groups related to the two streams of visual information processing, namely, the ventral and dorsal streams for object vision and spatial vision, respectively (Ungerleider and Mishkin, 1982). According to this proposal, the pulvinar nuclei provide cortico-pulvinar-cortical interactions that spread information both across areas within each visual stream and across streams, as well as relay information from the SC, via P3, largely to the dorsal stream areas.

There are two feedforward projections to V2, one from the lateral/inferior pulvinar and the other from V1. Inasmuch as neither the pulvinar nor V2 can be driven visually following V1 removal, either or both of these inputs to V2 could be drivers (Marion et al., 2013). Reversibly inactivating lateral pulvinar in the galago, a prosimian, prevented supragranular V1 neurons from responding to visual stimulation (Purushothaman et al., 2012). Reversible, focal excitation of lateral pulvinar receptive fields were found to increase the visual responses in coincident V1 receptive fields 4-fold and shift partially overlapping V1 receptive fields toward the center of excitation (Purushothaman et al., 2012). V1 responses to regions surrounding the excited lateral pulvinar receptive fields were suppressed. Excitation of lateral pulvinar after LGN lesions activated supragranular layer V1 neurons. If these results also hold in other primates, the lateral pulvinar would be in a powerful position to control and gate information outflow from V1 during changes of state or attention (Sherman and Guillery, 2002; Purushothaman et al., 2012).

Consistent with this role of the pulvinar in regulating effects of spatial attention in V4, deactivation of this portion of the pulvinar causes spatial attention deficits in monkeys (Desimone et al., 1990), and joint recordings in V4 and the lateral pulvinar show synchronized activity between the two structures, which is modulated by attention (Saalmann and Kastner, 2011).

Shipp (2003) reviewed the published data on the connectivity of the pulvinar with the cortex and its topography and he proposed a simplified, global model of the organization of cortico-pulvinar connections. According to this model, connections between the cortex and pulvinar are topographically organized and, as a result, the pulvinar contains four topographically ordered “maps.” The model also identified connection domains, and reconciles the coexistence of visual and cortical maps in two of them. Shipp (2003) proposed a replication principle of central-peripheral-central projections that operates at and below the level of domain structure. He hypothesized that cortico-pulvinar circuitry replicates the pattern of cortical circuitry but not its function, playing a more regulatory role instead. The cells of origin in V4 and their termination in the pulvinar suggest that the cortical-pulvinar-cortical connections define a pathway by which deep layer cells of cortical visual areas, via pulvinar, affect superficial layer cells of coupled cortical areas.

#### Claustrum

The claustrum is a thin, irregular, sheet-like neuronal structure hidden beneath the inner surface of the neocortex. Crick and Koch (2005) summarized what was known about the claustrum, and they speculated on its possible relationship to the processes that give rise to integrated conscious percepts. We found extensive reciprocal connections between V4 and the ventral portion of the claustrum (termed vCl), which extended through at least one-half of the rostrocaudal extent of the structure. Additionally, in about 75% of the cases, we found reciprocal connections between V4 and a more restricted region in the claustrum located farther dorsal, near the middle of the structure (termed mCl). Both vCl and mCl appear to have a crude topographic organization, based on the visuotopic location of our injection sites (Fig. 1). The portions of the claustrum connected with V4 appear to overlap considerably with those portions connected with other visual cortical areas, including V1 (Mizuno et al., 1981; Doty, 1983), V2 (Pearson et al., 1982), MT (Maunsell and Van Essen, 1983; Ungerleider et al., 1984), MST and FST (Boussaoud et al., 1992), TEO (Webster et al., 1993), and TE (Whitlock and Nauta, 1956; Kemp and Powell, 1970; Turner et al., 1980; Baizer et al., 1993; Webster et al., 1993). Evidence in other species suggests that the claustrum may be specialized for visuomotor tasks by virtue of its connections with different visual and motor subdivisions of cortex (Olson and Graybiel, 1980). Based primarily on findings from a study using 2-DG, Ettlinger and Wilson (1990) speculated that the claustrum is involved in cross-modal associations.

#### Amygdala

The connections between V4 and the amygdala were restricted to the dorsal portion of the lateral basal nucleus, where both retrogradely labeled cells and anterogradely labeled terminals were found in about 25% of our cases. This nucleus of the amygdala has also been found to be reciprocally connected with inferior temporal areas TE and TEO as well as with both perirhinal and parahippocampal cortices (Webster et al., 1993; Stefanacci et al., 1996; Stefanacci and Amaral, 2000, 2002; Chareyron et al., 2012). In an early study, Amaral and Price (1984) reported that the amygdala also projects to early extrastriate areas of the occipital lobe, including V1 and V2, suggesting an asymmetry in the inputs and outputs of this structure. This may also hold for V4; whereas 50% of our retrograde cases showed labeled cells in the amygdala projecting to V4, only 20% of our anterograde cases showed labeled terminals in the amygdala projecting from V4. The function of the amygdala projection to V4 may be to regulate cortical activity according to the emotional associations of visual objects (Pessoa and Adolphs, 2010), which would not require detailed retinotopic specificity.

#### Interlaminar layers of the LGN

In addition to visual pathways through the parvocellular and magnocellular layers of the lateral geniculate nucleus (LGN), a third parallel pathway exists, which projects to superficial layers I and III of striate cortex, originates in the koniocellular (interlaminar and S) layers of the LGN, and which are, in turn, rich in calbindin (Jones and Hendry, 1989; Tigges and Tigges, 1991; Casagrande, 1994; Johnson and Casagrande, 1995; Goodchild and Matin, 1998; Soares et al., 2001b). Calretinin-immunoreactive (Cr-IR) cells are concentrated in the S layers (Yan et al., 1996). These koniocellular layers receive projections from a population of small retinal ganglion cells (Itoh et al., 1982) and from small fibers originating in the superficial layers of the superior colliculus (May, 2006). We found labeling at the interlaminar layers of the lateral geniculate nucleus, but we failed to find cells from koniocellular layers of LGN to V4, as one of the authors (Soares et al., 2001a) previously reported. This result was somewhat surprising, as direct projections from the koniocellular layers of the LGN to area V4 have been reported previously by several groups (Wong-Riley, 1976; Benevento and Yoshida, 1981; Yoshida and Benevento, 1981; Yukie and Iwai, 1981; Bullier and Kennedy, 1983), as have projections from the koniocellular layers of the LGN to areas MT (Sincich et al., 2004) and TEO (Webster et al., 1993). It is very possible that we did not find those cells because they are small, and the fluorescent plotting was done after plotting the cortico-cortical projections, which may have caused the fluorescence to fade.

### Efferents of V4

#### Superior colliculus

The superficial layers of the superior colliculus receive direct retinotopically organized projections from the K and M ganglion cells in the retina, which are restricted to the upper half of the stratum griseum superficiale (Hendrickson et al., 1970; Ogren and Hendrickson, 1976; Graham, 1982). Whereas the projections from V1 to the superior colliculus are similarly restricted to the upper half of the stratum griseum superficiale (Ungerleider et al., 1984), those from extrastriate areas V2, MT, and TEO extend through this stratum to include the stratum opticum as well (Ungerleider et al., 1984; Webster et al., 1993). For both striate and extrastriate areas, projections to the colliculus are in register with the visuotopic organization of the structure (Cynader and Berman, 1972). This was also found to be true for the projections from area V4, which terminated in the same strata as projections from other extrastriate visual areas, namely, the stratum griseum superficiale and the stratum opticum. Inasmuch as visuotopic inputs to the colliculus are superimposed on an oculomotor map (Baleydier and Mauguiere, 1978; Wallace et al., 1997; Skaliora et al., 2004; Tabareau et al., 2007), it may be that projections from V4 provide visual feature information, which could trigger orienting oculomotor reactions to spatially localized based on unexpected form, color, or texture (Zénon and Krauzlis, 2012).

#### Thalamic reticular nucleus

In about half of the cases studied, we saw projections from V4 to the thalamic reticular nucleus. Unlike other “nonspecific” thalamic nuclei, the reticular nucleus projects not to cortex but rather to other thalamic nuclei. This projection is thought to provide an inhibitory feedback from cortex to thalamocortical neurons (Jones and Yang, 1985). The label in the reticular nucleus was too variable to determine its topographic organization.

### Striatum

#### Caudate nucleus

Kemp and Powell (1970) proposed that the cortical connections with the caudate nucleus obey a principle of “proximity,” with a given cortical region projecting to the portion of the caudate that was physically closest to it. According to this principle, the frontal cortex would project to the head of the caudate, the parietal cortex to the body, the occipital cortex to the genu, and the temporal cortex to the tail. However, subsequent studies showed that cortical projections are less topographic than originally proposed. For example, Selemon and Goldman-Rakiç (1985) demonstrated that cortical projections to the caudate nucleus terminate in elongated parasagittal strips rather than in discrete zones. In some cases, these strips appear to terminate through nearly the full length of the head and tail of the caudate (i.e., excluding the genu and parts of the body), whereas in other cases they seem to extend throughout the entire nucleus. This organizational scheme differs from that proposed by Saint-Cyr et al. (1990), who found that the projection strips arising from cortical visual areas are limited in length, and thus show some degree of topographic proximity. Similarly, Webster et al. (1993) found that the projections from inferior temporal areas TEO and TE follow the organization of elongated strips but are also characterized by topographic proximity. The present results are consistent with this organizational scheme, namely, elongated projection strips with some degree of topographic proximity. In our study, a crude visuotopic organization was also found: upper field V4 injections labeled the genu and the tail of the caudate, while lower field injections labeled mainly the head, body, and genu. Consistent with this topography, Hikosaka et al. (1989) and Yamamoto et al. (2012) recently reported that cells in the tail of the caudate combine visual object selectivity with visual-spatial specificity. This organization along the rostrocaudal dimension contrasts with that seen along the mediolateral dimension, in which interleaved projection zones confined to the dorsolateral head of the caudate were found.

#### Putamen

We found a projection from V4 to the putamen, which was restricted to its most caudal portion, where the nucleus appears to be segmented into small islands when viewed in coronal section. No visuotopic organization in the projections was found. The projection zone of V4 overlaps, but is somewhat posterior to and much less extensive than, that of areas TEO and TE (Saint-Cyr et al., 1990; Baizer et al., 1993; Webster et al., 1993); by contrast, the projection zone of V4 overlaps, but is somewhat anterior to and much more extensive than, that of area MT (Ungerleider et al., 1984). In general, comparable injections in V4, TEO, and TE show that the V4 projection is much smaller than those from TEO or TE (Webster et al., 1993).

The presence of projections from extrastriate visual cortical areas, such as V4, to the striatum, including both the caudate and putamen, suggests a possible involvement of mid- to high-level visual information in the control of complex motor behavior. Consistent with this idea, Rolls et al. (1983) found visually responsive cells in the caudate and putamen whose activity was dependent on the performance of the task and did not change in response to visual stimulation or hand movements, unless these were part of the task. Mishkin et al. (1984) and Mishkin and Appenzeller (1987) proposed that the caudate nucleus and the putamen form part of a circuit that receives visual information from high-level areas of the cortex (i.e., from inferior temporal cortex) and that is responsible for the formation of visuomotor associations, or visual “habits.” Support for this idea comes from lesions of the tail of the caudate nucleus and ventrocaudal putamen in monkeys that show deficits on discrimination learning of visual patterns (Divac et al., 1967; Buerger et al., 1974; Hikosaka and Wurtz, 1989).

## CONCLUSION

We previously studied the cortical connections of V4 and found a central-peripheral asymmetry in the projections to the temporal and the parietal cortices (Ungerleider et al., 2008). We concluded that peripheral field projections from V4 to parietal areas could provide a direct route for rapid activation of circuits serving spatial vision and spatial attention, while the predominance of central field projections from V4 to inferior temporal areas could provide the necessary information needed for detailed form analysis for object vision. In this study we studied the subcortical connections of V4 and found no evidence for central-peripheral asymmetry; instead, as shown in figure 15, we found both topographical and nontopographical projections to subcortical structures. These data led us to propose a segregation of topographical bidirectional projections to four fields of the pulvinar, to two subdivisions of the claustrum, and to the interlaminar portions of the lateral geniculate nucleus, structures that may operate as gates for spatial attention. The topographical efferent projections to the superficial and intermediate layers of the superior colliculus, the thalamic reticular nucleus, and the caudate nucleus suggest that these structures may also be involved in the processing of visual spatial attention.

## ROLE OF AUTHORS

Ricardo Gattass, Robert Desimone, and Leslie G. Ungerleider planned and executed the experiments, discussed the data, and wrote the article. Ricardo Gattass, Thelma W. Galkin, and Leslie G. Ungerleider plotted or reviewed the data and prepared the illustrations.

## Cortical sulci

amt: Anterior middle temporal sulcus
ar: Arcuate sulcus
ca: Calcarine fissure
ce: Central sulcus
ec: External calcarine sulcus
io: Inferior occipital sulcus
ip: Intraparietal sulcus
la: Lateral sulcus
lu: Lunate sulcus
ot: Occipitotemporal sulcus
p: Principal sulcus
po: Parietal occiptal cleft
pmt: Posterior middle temporal sulcus
rh: Rhinal sulcus
sp: Subparietal sulcus
st: Superior temporal sulcus

## Abbreviations

Amyg(ab): Accessory basal nucleus of amygdala
Amyg(l): Lateral nucleus of amygdala
Amyg(lb): Lateral basal nucleus of amygdala
Amyg(m): Medial nucleus of amygdala
Amyg(mb): Medial basal nucleus of amygdala
Br: Brachium of superior colliculus
Cd: Caudate nucleus
DR: Dorsal raphe
HA: Habenulla nucleus
HM: Horizontal meridian
II: Second ventricule
IT: Inferotemporal cortex
LG: Lateral geniculate nucleus
LG: I, intralaminar lateral geniculate nucleus
LC: Locus coeruleus
mCl: Medial claustrum
MD: Medium dorsal nucleus of the thalamus
MG: Medial geniculate nucleus
MR: Medial raphe
MT: Middle temporal area
nbM: Basal nucleus of Meynert
OT: Optic track
P1-P4: Subdivisions of pulvinar
PI: Inferior pulvinar
PL: Lateral pulvinar
PM: Medial pulvinar
Pul: Pulvinar
Put: Putamen
R: Thalamic reticular formation
SC: Superior colliculus
SG: Supra geniculate nucleus
TEO: Visual area TEO
Thal: Thalamus
V1: Primary visual cortex
V2: Second visual area
V4: Visual area four
vCl: Ventral claustrum
VT: Ventral tegmentum
